# Prevalence and associated factors of impaired kidney functions among children and adolescents in India: insights from the Comprehensive National Nutrition Survey (CNNS) 2016-18

**DOI:** 10.1186/s12887-024-04903-y

**Published:** 2024-07-04

**Authors:** Angad Singh, Madhur Verma, Milan Das, Pragyan Paramita Parija, Saurabh Nayak, Vivekanand Jha

**Affiliations:** 1 International Institute for Population Sciences, Mumbai, India; 2 Community and Family Medicine, All India Institute of Medical Sciences Bathinda, Punjab, India; 3 Community and Family Medicine, All India Institute of Medical Sciences Vijaypur, Jammu, India; 4 Nephrology, All India Institute of Medical Sciences Bathinda, Punjab, India; 5 The George Institute for Global Health India, UNSW, New Delhi, India; 6 Manipal Academy of Higher Education, Manipal, India; 7 Faculty of Medicine, Imperial College London, London, UK; 8 Faculty of Medicine, University of New South Wales, Sydney, Australia

**Keywords:** CNNS, Chronic Kidney Disease, Noncommunicable diseases, Child health, Sustainable development goals

## Abstract

**Background:**

Chronic kidney disease (CKD) is a significant public health problem. The burden of CKD in children and adolescents in India is not well described. We used data from the recent Comprehensive National Nutrition Survey (CNNS) to estimate the prevalence of impaired kidney function (IKF) and its determinants in children and adolescents between the ages of 5 and 19.

**Methods:**

CNNS 2016–18 adopted a multi-stage sampling design using probability proportional to size sampling procedure after geographical stratification of urban and rural areas. Serum creatinine was tested once in 24,690 children and adolescents aged 5–19 years. The estimated glomerular filtration rate (eGFR) was derived using the revised Schwartz equation. The eGFR value below 60 ml/min/1.73 m^2^ is defined as IKF. Bivariate analysis was done to depict the weighted prevalence, and multivariable logistic regression examined the predictors of IKF.

**Results:**

The mean eGFR in the study population was 113.3 + 41.4 mL/min/1.73 m^2^. The overall prevalence of IKF was 4.9%. The prevalence in the 5–9, 10–14, and 15–19 year age groups was 5.6%, 3.4% and 5.2%, respectively. Regression analysis showed age, rural residence, non-reserved social caste, less educated mothers, Islam religion, children with severe stunting or being overweight/obese, and residence in Southern India to be predictors of IKF.

**Conclusions:**

The prevalence of IKF among children and adolescents in India is high compared to available global estimates. In the absence of repeated eGFR-based estimates, these nationally representative estimates are intriguing and call for further assessment of socio-demographic disparities, genetics, and risk behaviours to have better clinical insights and public health preparedness.

**Supplementary Information:**

The online version contains supplementary material available at 10.1186/s12887-024-04903-y.

## Introduction

Chronic kidney disease (CKD), characterised by a reduced estimated glomerular filtration rate (eGFR), increased urinary albumin excretion, or both, is acknowledged as a pressing public health issue [1]. In addition to the risk of development of kidney failure, there is an inverse relationship between cardiovascular disease risk and eGFR independent of other known risk factors [2]. Therefore, the burden due to CKD needs active redressal to meet the United Nations Sustainable Development Goal target to reduce premature mortality from non-communicable diseases by a third by 2030 [3]. The Global Burden of Disease (GBD) study estimated that the all-age prevalence and mortality due to CKD increased by 29.3% and 41.5% between 1990 and 2017, respectively [4]. In Indian adults (18 and 70 years), the increase was up to 38% between 2001–03 and 2010–13 [5]. CKD has been recently included in the National Program for Non-Communicable Disease (NP-NCD).

Children constitute a small proportion of the CKD population but cannot be ignored due to long-term comorbidities. Globally, the prevalence of CKD in the 5–19 years age group is estimated to be around 98.1(85.0–114.43) cases per million, and the incidence is reported to be around 0.30(0.19–0.42) cases per million [6]. In other studies, the incidence has varied from 7.7 per million in Sweden to 74.7 per million in Italy, with creatinine clearance (C_Creatinine_) cut-off defined at < 30 and < 75 mL/min per 1.73 m^2^, respectively [7, 8]. In India, available evidence informs us that CKD is reaching epidemic proportions in specific geographical areas and tends to afflict relatively younger adults [9]. Epidemiological data on CKD in India are limited to small studies from regional pockets [5, 10], have not followed the current guidelines for diagnosis and classification [11, 12] and have focused on the late disease stages [13]. Two hospital-based studies estimated the prevalence of CKD in children to be 9.3–12%, using a C_Creatinine_ of < 50 ml/min [14–16].

The recent Indian Comprehensive National Nutrition Survey (CNNS) collected data on serum creatinine levels and offers an opportunity to estimate the eGFR values and the prevalence of impaired kidney function (IKF) among children and adolescents (age 5–19 years) [17]. Such estimates would be close to the actual estimates of CKD, and insights generated can be generalised to the children and in the country. In this manuscript, we present national-level estimates of IKF among children and adolescents aged 5–19 years and identify associated socio-demographic parameters using the data from CNNS.

## Methodology

### Data Source

This secondary data analysis used a nationally representative dataset generated from the CNNS carried out by the Ministry of Health and Family Welfare (MoHFW), Government of India, between 2016 and 2018 [17, 18]. The study selected a representative sample of households and individuals aged 0–19 across 30 states. The survey collected detailed information on participants' nutritional status, anthropometric markers, food intake, and micronutrient levels. The survey adopted a multi-stage sampling design after geographical stratification of urban and rural areas to select the primary sampling units. A two-stage sampling strategy was adopted for smaller primary sampling units (PSUs): in the first stage, PSUs were selected using a probability proportional to size (PPS) sampling, and in the second stage, a systematic random selection of households was made within each PSU. In large PSUs, the sampling design involved three stages, with the addition of a segmentation procedure to reduce enumeration areas to manageable sizes.

### Study population

CNNS provides comprehensive nutritional profiling of preschoolers (0–4 years), children of school-going age (5–9 years), and adolescents (10–19 years). Individuals in the chosen households were excluded if they had a significant physical deformity (e.g., paralysis, cerebral palsy) or cognitive disabilities, had an acute febrile or infectious illness, had a known chronic systemic illness, including tuberculosis, cancer, liver disease, and renal disease, were on medications for chronic conditions, had an acute injury, or were pregnant Currently married adolescents aged 10–19 years not visibly pregnant were asked whether they had a menstrual period in the past 30 days. If not, an hCG urine test was performed to confirm pregnancy [17, 18].

### Sample selection

**Fig. 1:**
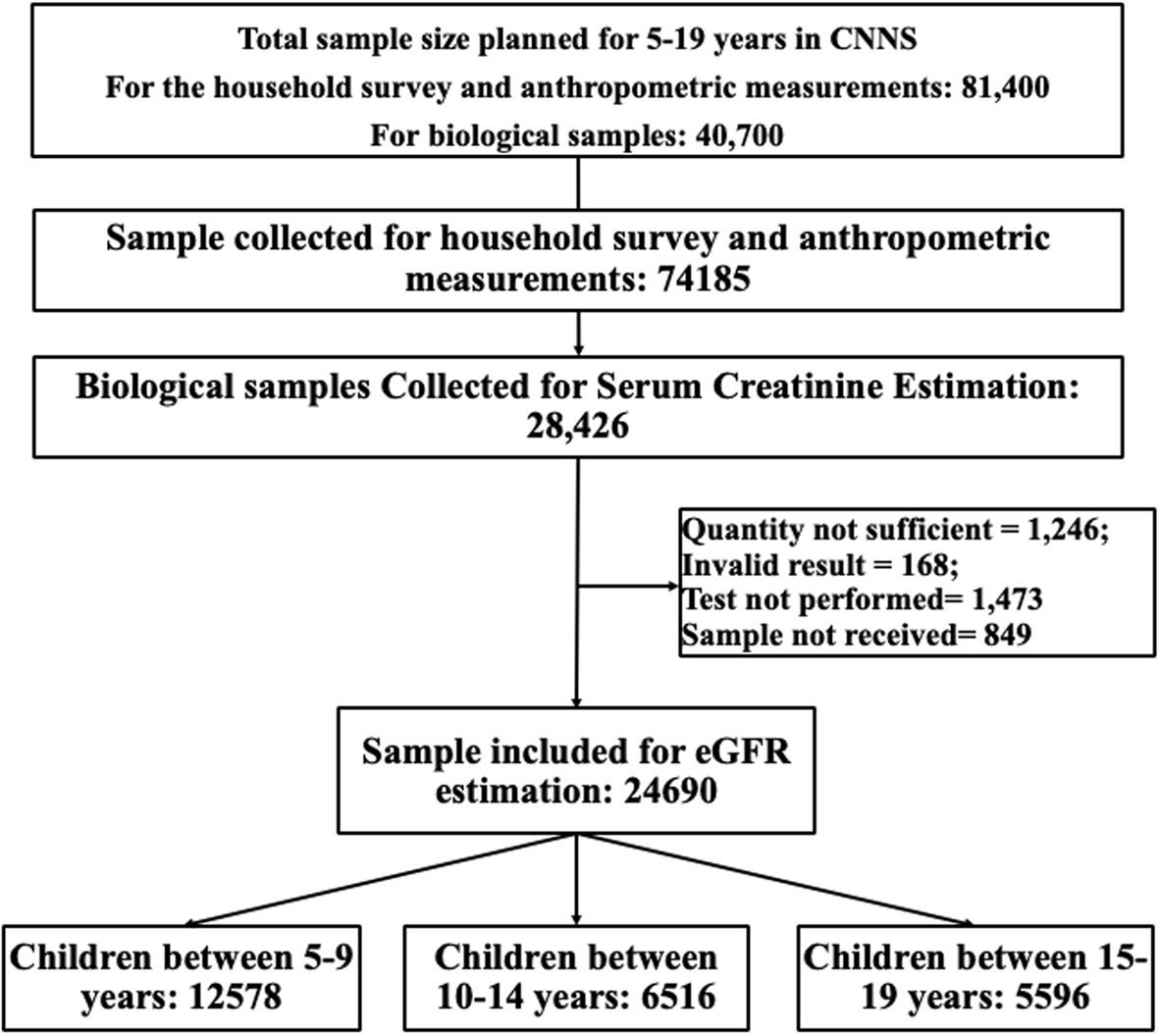
Sample selection flow chart for the present analysis using data from the Comprehensive National Nutrition Survey (CNNS) 2016–18

The survey originally included 122,100 children and adolescents (1–19 years). Due to a growing risk of non-communicable diseases in India, the renal function testing was limited to children aged 5 to 9 years and adolescents aged 10–19 years with a combined sample size of 74,185, and children between 1 and 4 years were excluded (Fig. 1). Further, we only included survey participants (*n* = 28,426) with available serum creatinine (sCr) information. Of these, 849 samples could not be processed, tests could not be performed in 1473, sample volume was insufficient for 1246, and 168 returned invalid results. Valid sCr was available for 24,690 participants, who were included in the final analysis.

### Blood sample collection

Blood draws were conducted by trained phlebotomists. Phlebotomists visited selected households and obtained the consent of the parent/caregiver of the child/adolescent, following which container for urine collection was provided. The next morning,10 millilitres (ml) of fasting blood sample was collected. All samples were labelled with a unique ID and barcoded. Serum creatinine was tested by Jaffe’s Method calibrated against standard reference materials.

### Study variables

#### Dependent variable

Kidney function was assessed based on Wallach’s Interpretation of Diagnostic Tests 2013 guidelines [19]. The eGFR was estimated by using the Revised Schwartz equation *(Estimated GFR, mL/min/1.73 m2* = *k* × *height, cm/serum creatinine, mg/dL; where k* = *0.413 *[19] as recommended by Kidney Disease Improving Global Outcomes (KDIGO) guidelines. Individuals with eGFR below 60 ml/min/1.73 m2 were classified as having IKF [20, 21].

#### Independent variables

The variables were chosen based on a comprehensive literature review and segregated at the level of child, mother and household [2, 5, 22, 23]. We included age (5 –9, 10 – 14, and 15 – 19 years), residence (Rural and Urban), religious groups (Hindu, Islam, and Others), caste (Schedule cast, Schedule Tribe, Other Backward Class (OBC), and non-reserved), education status (Ever attended School: Yes, No), Wealth Index (Poorest, Poorer Middle, Richer, and Richest), mother’s education (No education, < 5 years, 5–8 years, 9–11 years, and ≥ 12 years of schooling), type of diet (Vegetarian, Vegetarian with egg, Non-vegetarian), Stunting, BMI-for-age was classified as per the definitions used in the CNNS. The height-for-age z-score below minus 3SD, and minus 2SD below the median on the WHO Child Growth Standards depicted severe or moderate stunting [24]. The BMI-for-age > + 1SD and > + 2SD above the WHO Child Growth Standards median depicted overweight and obesity, while values < -2SD were labelled as thinness [24]. Regions of India (North, Central, East, Northeast, West, and South) were categorised as per a previously published study using the same dataset [22].

#### Ethics and consent/assent procedure

Ethics approvals was obtained from the Institutional Review Board (IRB) of the Population Council and the Postgraduate Institute of Medical Education and Research Chandigarh Institutional Ethics Committee. For children aged 0–17 years, informed consent was obtained from parents/caregivers, in addition to assent from adolescents aged 11–17 years, and only informed consent from adolescents aged 18–19 years [17]. Parents/caregivers of children /adolescents identified with critical conditions from the analysis of the biological samples were given an immediate alert. The survey laboratories developed the methods to identify critical conditions (Critical Call-Out) for conditions like diabetes, high blood pressure, CKD, severe anaemia and other conditions. The test results were provided in a sealed envelope, along with a general information sheet guiding parents/caregivers on whom to consult for further care and management.

#### Statistical analysis

Data were analysed using STATA 16. The sCr and eGFR were compared within age groups using unpaired t-test and ANOVA. The association between abnormal eGFR and socio-demographic variables was examined using bivariate analysis. A logistic regression model was used to identify factors that predicted IKF. The factors included in the final model were based on a literature review, and those found significant on bivariate analysis. The unadjusted and adjusted odds ratios depicting the likelihood of IKF in the population were adjusted to consider the complex CNNS 2016–18 sampling design by including the primary sampling units, sampling weights, and strata in the models. We used Microsoft Excel maps to graphically depict the prevalence of children with abnormal kidney functions.

## Results

Table 1 depicts the background characteristics of the children in the three age groups. The representation of the males was a little higher (52.3%) than the females. More children were from rural areas (57.3%), and a higher proportion belonged to the Hindu religion (72.6%) and other backward social classes (33.4%). Nearly one-third were from the wealthiest social strata (33.9%), and most (95.8%) attended school. About 30% of the mothers had never been to school, and nearly two-fifths of the children (79.4%) consumed a non-vegetarian diet. The prevalence of stunting was around 20%, being highest in 15–19-year-old age groups. The overall prevalence of thinness (low BMI for age < -2SD) was 79% and was highest in the youngest group (95.3%).

**Table 1:** Sample characteristics of the children and adolescents (05–19 years) who participated in the CNNS, 2016–18, India

Background characteristics	5–9 years	10–14 years	15–19 years	Total
	**Unweighted counts (Proportions)**	**Unweighted counts (Proportions)**	**Unweighted counts (Proportions)**	**Unweighted counts (Proportions)**
**Total**	**12,578 (52.4)**	**6516 (27.2)**	**5596 (20.4)**	**24,690**
**Child level variables**				
** Sex of the child**				
Male	6687 (53.2)	3413 (52.4)	2806 (50.1)	12,906 (52.3)
Female	5891 (46.8)	3103 (47.6)	2790 (49.9)	11,784 (47.7)
**Stunting**				
Not present	10,139 (82.3)	4979 (77.7)	3541 (73.9)	18,659 (79.3)
Moderate	1662 (13.5)	1035 (16.2)	989 (20.6)	3686 (15.7)
Severe	518 (4.2)	394 (6.2)	263 (5.5)	1175 (5.0)
**BMI-for-age**				
Thin	11,728 (95.3)	4923 (77.1)	2431 (44.5)	19,082 (79.0)
Healthy	495 (4.0)	1303 (20.4)	2689 (49.3)	4487 (18.6)
Over Weight/Obese	78 (0.6)	157 (2.5)	339 (6.2)	574 (2.4)
**Ever attended School**				
Yes	11,851 (94.2)	6382 (97.9)	5419 (96.8)	23,652 (95.8)
No	727 (5.8)	134 (2.1)	177 (3.2)	1038 (4.2)
**Type of Diet**				
Vegetarian	2008 (16.0)	1016 (15.6)	997 (17.8)	4021 (16.3)
Vegetarian with egg	570 (4.5)	266 (4.1)	221 (4.0)	1057 (4.3)
Non-vegetarian	10,000 (79.5)	5234 (80.3)	4378 (78.2)	19,612 (79.4)
**Mother’s level variables**				
** Mother's education**				
No education	3173 (25.3)	2196 (34.1)	2199 (39.5)	7568 (30.8)
< 5 years completed	1055 (8.4)	532 (8.3)	497 (8.9)	2084 (8.5)
5–8 years completed	3209 (25.6)	1624 (25.2)	1286 (23.1)	6119 (24.9)
9–11 years completed	2727 (21.8)	1235 (19.2)	1030 (18.5)	4992 (20.3)
≥ 12 years completed	2370 (18.9)	856 (13.3)	550 (9.9)	3776 (15.4)
**Household level variables**				
** Residence**				
Rural	7183 (57.1)	3741 (57.4)	3218 (57.5)	14,142 (57.3)
Urban	5395 (42.9)	2775 (42.6)	2378 (42.5)	10,548 (42.7)
**Religious groups**				
Hindu	9115 (72.5)	4689 (72.0)	4123 (73.7)	17,927 (72.6)
Islam	1449 (11.5)	763 (11.7)	594 (10.6)	2806 (11.4)
Others	2014 (16.0)	1064 (16.3)	879 (15.7)	3957 (16.0)
**Social Caste**				
Non-reserved	3188 (26.6)	1690 (27.3)	1447 (27.2)	6325 (26.9)
Schedule cast	2495 (20.9)	1316 (21.2)	1114 (20.9)	4925 (21.0)
Schedule tribe	2258 (18.9)	1191 (19.2)	948 (17.8)	4397 (18.7)
Other Backward class	4028 (33.7)	2002 (32.3)	1819 (34.1)	7849 (33.4)
**Wealth Index**				
Poorest	893 (7.1)	573 (8.8)	401 (7.2)	1867 (7.6)
Poorer	1464 (11.6)	862 (13.2)	681 (12.2)	3007 (12.2)
Middle	2383 (19.0)	1271(19.5)	1087 (19.4)	4741 (19.2)
Richer	3502(27.8)	1684(25.8)	1520 (27.2)	6706 (27.2)
Richest	4336(34.5)	2126(32.6)	1907 (34.1)	8369 (33.9)
**Regions of India**				
North	3023 (24.0)	1562 (24.0)	1432 (25.6)	6017 (24.4)
Central	1305 (10.4)	664 (10.2)	534 (9.5)	2503 (10.1)
East	2403 (19.1)	1228 (18.9)	1056 (18.9)	4687 (19.0)
Northeast	2808 (22.2)	1517 (23.3)	1195 (21.4)	5520 (22.4)
West	1203 (9.6)	605 (9.3)	549 (9.8)	2357 (9.6)
South	1836 (14.6)	940 (14.4)	830 (14.8)	3606 (14.6)

**Fig. 2:**
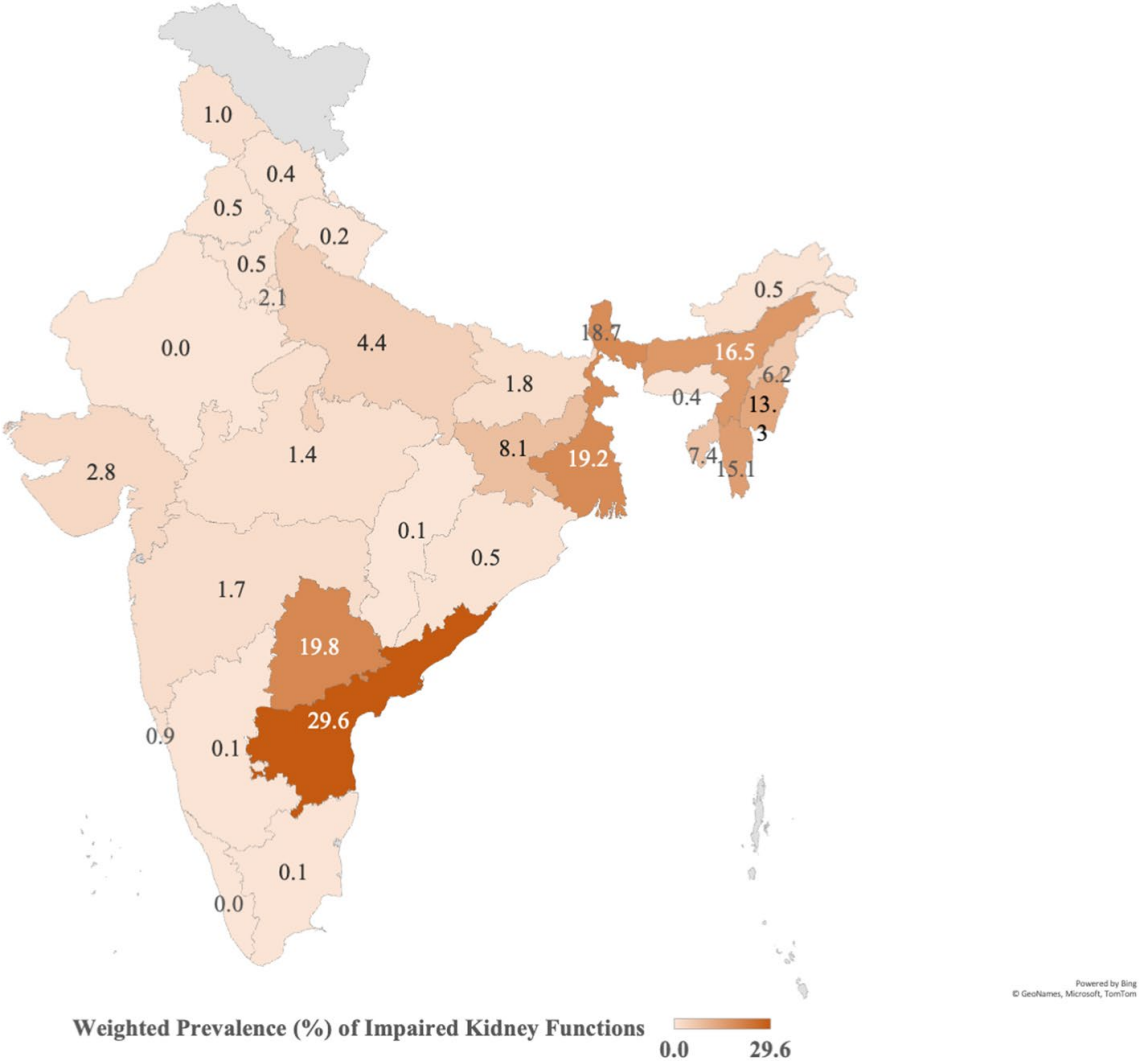
State-wise prevalence of impaired kidney function in children and adolescents per the Comprehensive National Nutrition Survey (CNNS) 2016–18

Supplementary Table 1 depicts the age-wise distribution of the study population as per their eGFR values. Table 2 further depicts the eGFR values amongst the various subgroups. The mean eGFR was 113.3 + 41.4 mL/min/1.73 m^2^. The eGFR was highest in 5–9 years (117.5 ± 44.1) and decreased with increasing age. The eGFR varied significantly across gender, religion, social castes, wealth index, mother’s education, type of diet, stunting, BMI-for-age, and regions. Table 3 shows the prevalence of IKF. The prevalence was 4.9% (95% CI: 4.7–5.2) overall and 5.6% (5.2–6.0), 3.4%(3.0–3.9) and 5.2%(4.7–5.9) in the 5–9, 10–14, and 15–19 year age groups respectively. In 5–9 years, prevalence varied significantly across all the studied independent variables except gender, while in 10–14 years, the differences were non-significant across gender, attending schools, mother’s education, and BMI-for-age. In the 15–19 years, the prevalence varied significantly across gender, social caste, wealth index, mother’s education, type of diet, height-for-age, BMI-for-age, and regions of India. Figure 2 depicts the prevalence of IKF across different states and union territories of India. The highest prevalence was observed in Andhra Pradesh, followed by Telangana and West Bengal, while the prevalence was lowest in Tamil Nadu, Chhattisgarh, Rajasthan and Kerala.

**Table 2:** Comparison of the mean eGFR values (*ml/min/1.73 m*^*2*^) within three age groups per the socio-demographic characteristics included in the CNNS, 2016–18, India

Background characteristics	5–9 years		10–14 years		15–19 years		Total	
	**Mean (± SD)**	***p***-value*	**Mean (± SD)**	*p*-value*	**Mean (± SD)**	*p*-value*	**Mean (± SD)**	*p*-value*
**Sample Size**	**12,578**		**6516**		**5596**		**24,690**	
**Overall mean eGFR**	**117.5 (44.1)**		**116.5 (41.1)**		**99.6 (31.4)**		**113.2 (41.4)**	
**Child level variables**								
**Sex of the child**		< 0.001		< 0.001		< 0.001		< 0.001
Male	115.3 (42.1)		111.6 (40)		92.7 (29.6)		109.4 (40.1)	
Female	120.0 (46.2)		121.8 (41.7)		106.6 (31.7)		117.3 (42.4)	
**Stunting**		< 0.001		< 0.001		< 0.001		< 0.001
Not present	119.1 (43.3)		118.2 (40.8)		101.4 (29.6)		115.5 (40.9)	
Moderate	114.9 (43.8)		114.9 (38.6)		99.1 (31.8)		110.7 (40.0)	
Severe	105.2 (47.8)		98.7 (47.3)		83.2 (41.1)		98.1 (46.9)	
**BMI for age**		< 0.001		< 0.001		< 0.001		< 0.001
Thin	118.1 (43.4)		118.2 (41.0)		102.1 (30.7)		116.1 (41.8)	
Healthy	118.8 (44.3)		113.8 (37.9)		98.8 (30.4)		105.4 (35.4)	
Over Weight/Obese	87.0 (57.2)		100.2 (42.4)		97.2 (31.2)		96.6 (39.0)	
**Type of Diet**		< 0.001		0.108		< 0.001		< 0.001
Vegetarian	121.7 (43.1)		118.7 (34.9)		105.4 (28.2)		116.9 (38.5)	
Vegetarian with egg	122.8 (44.8)		118.6 (35.9)		108.3 (35.3)		118.7 (41.2)	
Non-vegetarian	116.4 (44.2)		115.9 (42.5)		97.9 (31.7)		112.1 (42.0)	
**Mother’s level variable**								
**Mother's education**		0.003		0.296		0.387		< 0.001
No education	115.3 (44.8)		114.9 (41.7)		99.5 (32.7)		110.6 (41.0)	
< 5 years completed	118.5 (46.9)		117.6 (41.9)		98.5 (29.0)		113.5 (42.8)	
5–8 years completed	116.3 (45.0)		117.3 (42.5)		100.8 (31.8)		113.3 (42.4)	
9–11 years completed	119.4 (44.9)		117.4 (41.5)		98.6 (31.2)		114.6 (42.4)	
≥ 12 years completed	119.7 (40.5)		117.0 (36.0)		100.6 (27.9)		116.3 (38.4)	
**Household level variables**								
**Residence**		< 0.001		0.002		0.502		< 0.001
Rural	116.1 (44.7)		115.1 (41.4)		99.4 (32.1)		112 (41.9)	
Urban	119.4 (43.2)		118.3 (40.6)		100.0 (30.5)		114.8 (40.8)	
**Ever attended School**		0.219		0.158		0.512		0.208
Yes	117.4 (43.8)		116.6 (41.1)		99.7 (31.5)		113.1 (41.2)	
No	119.5 (49.3)		111.5 (42.5)		98.1 (29.0)		114.8 (46.3)	
**Religious groups**		0.019		0.799		0.032		0.037
Hindu	117.4 (43.3)		116.2 (40.2)		100.2 (31.2)		113.1 (40.6)	
Islam	115.3 (45.0)		116.8 (40.2)		96.6 (31.4)		111.7 (41.9)	
Others	119.6 (47.0)		117.1 (45.5)		99.1 (32.4)		114.4 (44.5)	
**Social Caste**		< 0.001		0.024		0.526		< 0.001
Non-reserved	119.2 (45.1)		118.4 (44.2)		100.9 (32.4)		114.8 (43.0)	
Schedule cast	114.6 (42.9)		114.2 (35.8)		99.7 (29.9)		111.1 (38.5)	
Schedule tribe	119.9 (47.6)		118.4 (47.9)		99.0 (33.5)		115.0 (45.8)	
Other Backward class	118.4 (41.2)		116.6 (37.1)		100.3 (30.8)		113.8 (38.6)	
**Wealth Index**		< 0.001		0.138		0.851		< 0.001
Poorest	111.1 (44.5)		115.1 (48.3)		99.8 (35.5)		109.9 (44.3)	
Poorer	114.0 (44.1)		113.5 (39.1)		98.5 (33.8)		110.3 (41.1)	
Middle	117.0 (47.7)		116.6 (42.8)		99.4 (31.9)		112.9 (43.8)	
Richer	117.6 (44.4)		116.7 (40.7)		100.0 (31.8)		113.4 (41.5)	
Richest	120.2 (41.5)		117.7 (39.0)		99.8 (29.0)		114.9 (39.2)	
**Regions of India**		< 0.001		< 0.001		< 0.001		< 0.001
North	123.6 (42.0)		122.9 (39.7)		105.4 (30.9)		119.1 (39.8)	
Central	114.1 (37.1)		115.4 (33.5)		100.5 (28.4)		111.5 (34.9)	
East	110.8 (43.2)		111.9 (43.2)		96.7 (32.1)		107.9 (41.4)	
Northeast	117.4 (52.5)		114.8 (48.1)		96.1 (35.5)		112.1 (48.8)	
West	130.0 (42.8)		123.5 (36.8)		105.6 (29.6)		122.7 (39.8)	
South	110.2 (36)		110.4 (33.4)		94.0 (25.6)		106.5 (33.9)	

^*^p-value calculated using unpaired student’s test for independent variables with two categories and one-way ANOVA for variables with ≥ 3 categories

**Table 3:** Prevalence of impaired kidney functions within three age groups per the socio-demographic characteristics included in the CNNS, 2016–18, India

Background characteristics	5–9 years		10–14 years		15–19 years		Total	
	**Weighted prevalence** **(95% CI)**	*P*-Value*	**Weighted prevalence** **(95% CI)**	*P*-Value*	**Weighted prevalence** **(95% CI)**	*P*-Value*	**Weighted prevalence** **(95% CI)**	*P*-Value*
**Sample size**	**12,578**		**6516**		**5596**		**24,690**	
**Total**	**5.6 (5.2–6.0)**		**3.4 (3.0–3.9)**		**5.2 (4.7–5.9)**		**4.9 (4.7–5.2)**	
**Child level variables**								
**Sex of the child**		0.131		0.380		0.002		0.451
Male	5.1 (4.5–5.7)		3.8 (3.2–4.5)		6.0 (5.2–7.0)		4.9 (4.5–5.3)	
Female	6.2 (5.6–6.8)		2.9 (2.4–3.6)		4.5 (3.8–5.4)		4.9 (4.5–5.3)	
**Stunting**		< 0.001		0.004		< 0.001		< 0.001
**Not present**	5.2 (4.7–5.6)		2.7 (2.3–3.3)		3.4 (2.9–4.1)		4.16 (3.9–4.5)	
**Moderate**	7.1 (5.9–8.4)		5.5 (4.3–6.9)		8.4 (6.9–10.2)		6.9 (6.2–7.8)	
**Severe**	7.9 (6.0–10.3)		4.5 (2.8–7.1)		17.7 (13.7–22.6)		9.1 (7.6–10.8)	
**BMI-for-age**		< 0.001		0.071		0.0390		< 0.001
Thin	5.6 (5.2–6.0)		3.2 (2.8–3.7)		5.9 (5.1–6.9)		4.9 (4.7–5.3)	
Healthy	3.3 (1.6–6.7)		4.2 (3.0–5.8)		4.3 (3.6–5.2)		4.2 (3.6–4.9)	
Over Weight/Obese	17.7 (8.6–32.9)		6.1 (2.7–12.9)		6.3 (3.7–10.6)		7.6 (5.2–10.9)	
**Ever attended School**		0.007		0.303		0.306		< 0.001
Yes	5.38 (5.0–5.8)		3.5 (3.0–4.0)		4.9 (4.4–5.6)		4.8 (4.5–5.1)	
No	8.14 (6.5–10.1)		1.1 (0.3–4.0)		9.4 (6.6–13.3)		7.4 (6.2–8.9)	
**Type of Diet**		< 0.001		< 0.001		< 0.001		< 0.001
Vegetarian	3.4 (2.7–4.2)		0.6(0.3–1.2)		2.4 (1.7–3.5)		2.4 (2.0–2.9)	
Vegetarian with egg	1.4 (0.6–3.0)		2.2 (1.1–4.4)				1.3 (0.8–2.2)	
Non-vegetarian	6.4 (5.9–6.9)		4.25 (3.7–4.9)		6.4 (5.7–7.3)		5.9 (5.5–6.2)	
**Mother’s level variables**								
**Mother's education (completed)**		< 0.001		0.083		0.003		< 0.001
No education	6.3 (5.6–7.0)		3.63 (3.0–4.3)		6.2 (5.4–7.9)		5.5 (5.1–6.0)	
< 5 years	6.8 (5.4–8.6)		5.63 (3.9–8.1)		6.8 (4.7–9.9)		6.5 (5.5–7.7)	
5–8 years	5.5 (4.7–6.4)		2.8 (2.0–3.7)		4.3 (3.3–5.6)		4.5 (4.0–5.1)	
9–11 years	5.4 (4.5–6.5)		3.1 (2.1–4.6)		3.4 (2.2–5.2)		4.5 (3.8–5.3)	
≥ 12 years	3.1 (2.3–4.1)		2.02 (1.1–3.7)		0.9 (0.3–3.1)		2.6 (2.0–3.3)	
**Household level variables**								
**Residence**		< 0.001		< 0.001		0.353		< 0.001
Rural	6.3 (5.8–6.9)		3.8 (3.3–4.4)		6.2 (5.5–6.9)		5.6 (5.3–6.0)	
Urban	3.4 (2.8–4.1)		1.9 (1.3–2.7)		2.6 (1.9–3.6)		2.7 (2.4–3.3)	
**Religious groups**		< 0.001		< 0.001		0.222		0.001
Hindu	5.1 (4.7–5.6)		2.4 (2.0–2.9)		4.7 (4.1–5.4)		4.3 (4.0–4.6)	
Islam	7.2 (6.1–8.5)		6.3(4.9–8.1)		8.0 (6.3–10.2)		7.1 (6.3–8.1)	
Others	9.7 (7.3–12.7)		11.1 (8.0–15.4)		6.8 (4.1–11.1)		9.5 (7.8–11.5)	
**Social Caste**		< 0.001		< 0.001		< 0.001		< 0.001
Non-reserved	6.8 (5.9–7.8)		4.9 (4.0–6.2)		6.5 (5.3–8.0)		6.2 (5.6–6.9)	
Schedule cast	6.6 (5.7–7.6)		3.0 (2.2–4.1)		4.6 (3.6–5.9)		5.2 (4.6–5.8)	
Schedule tribe	6.7 (5.4–8.1)		5.1 (3.6–7.2)		11.0 (8.3–14.4)		7.0 (6.1–8.1)	
Other Backward class	3.6 (3.1–4.2)		2.1 (1.6–2.7)		3.6 (2.9–4.4)		3.2 (2.9–3.6)	
**Wealth Index**		< 0.001		< 0.001		< 0.001		< 0.001
Poorest	5.5 (4.6–6.5)		4.9 (3.8–6.2)		6.6 (5.2–8.5)		5.6 (4.9–6.3)	
Poorer	7.9 (6.9–9.0)		3.5 (2.6–4.6)		8.3 (6.7–10.2)		6.7 (6.0–7.4)	
Middle	7.3 (6.3–8.4)		2.6 (1.8–3.6)		6.0 (4.8–7.6)		5.8 (5.2–6.5)	
Richer	4.8 (4.0–5.6)		2.7 (1.9–3.9)		4.6 (3.5–5.9)		4.2 (3.7–4.9)	
Richest	2.2 (1.7–2.9)		3.1 (2.2–4.2)		1.3 (0.8–2.2)		2.2 (1.8–2.7)	
**Regions of India**		< 0.001		< 0.001		< 0.001		< 0.001
North	0.7 (0.4–1.4)		0.7 (0.3–1.7)		0.1 (0.0–1.2)		0.6 (0.4–1.0)	
Central	4.6 (3.9–5.4)		1.3 (0.9–1.9)		3.9 (3.07–4.9)		3.5 (3.1–4.0)	
East	7.8 (6.9–8.8)		4.6 (3.8–5.8)		9.5 (8.01–11.1)		7.3 (6.7–8.0)	
Northeast	13.7 (10.3–18.0)		18.7 (13.3–25.6)		7.3 (3.87–13.2)		13.6 (11.1–16.6)	
West	2.8 (2.0–3.9)		1.3 (0.7–2.8)		1.5 (0.7–3.0)		2.2 (1.6–2.9)	
South	7.7 (6.6–9.1)		6.0 (4.7–7.7)		7.5 (6.0–9.5)		7.2 (6.4–8.1)	

^*^p-value calculated using chi-square test

Table 4 presents the unadjusted and adjusted odds ratio and 95% CI for IKF with different independent variables. At the time of model building, we removed variables like ‘Ever attended School’, ‘Type of Diet’, and ‘BMI-for-age’ of the child as they depicted multi-collinearity. Adjusted analysis depicted the lower likelihood of having IKF in older age groups compared to the youngest (aOR:0.6; 95% CI: 0.5–0.7) and (aOR:0.8; 0.7–0.9), residence in urban areas (aOR:0.7; 0.6–0.8) compared to the rural, in other backward classes compared to the non-reserved social caste (aOR:0.6; 0.5–0.7), and having mothers with more years of education compared to no education (aOR: 0.7; 0.6–0.9). The likelihood of IKF was higher in moderate (aOR: 1.2; 1.1–1.4) and severe stunting (aOR: 1.8; 1.4–2.3), and residence in Indian regions other than the north, with the Southern region depicting the highest odds (aOR: 13.2; 9.3–18.7), respectively. Variables like religious groups and wealth index did not depict any significant OR.

**Table 4:** Unadjusted and adjusted risk factors of impaired kidney function in Indian children and adolescents per CNNS, 2016–18

Background characteristics	Unadjusted OR (95% CI)	*p*-Value	Adjusted OR (95% CI)	*p*-value
**Child level variables**				
** Age groups**				
05–09	Ref		Ref	
10–14	0.6 (0.55–0.74)	< 0.001	0.6 (0.5–0.7)	< 0.001
15–19	8.3 (0.72–0.95)	0.012	0.8 (0.7–0.9)	< 0.01
**Sex of the child**				
Male	Ref		-	-
Female	0.9 (0.85–1.07)	0.451	-	-
**Stunting**				
Not present	Ref		Ref	
Moderate	1.4 (1.2–1.6)	< 0.001	1.2 (1.1–1.4)	< 0.05
Severe	2.3 (1.8–2.8)	< 0.001	1.8 (1.4–2.3)	< 0.001
**Mother’s level variables**				
** Mother's education**				
No education	Ref		Ref	
< 5 years completed	1.1 (0.87–1.32)	0.504	0.8 (0.66–1.06)	0.144
5–8 years completed	0.9 (0.81–1.10)	0.473	1.0 (0.8–1.2)	0.901
9–11 years completed	0.7 (0.63–0.89)	0.001	0.7 (0.6–0.9)	< 0.01
≥ 12 years completed	0.6 (0.51–0.77)	< 0.001	0.7 (0.6–0.9)	< 0.05
**Household level variables**				
** Residence**				
Rural	Ref		Ref	
Urban	0.7 (0.60–0.77)	< 0.001	0.7 (0.6–0.8)	< 0.001
**Religious groups**				
Hindu	Ref		Ref	
Islam	1.1 (0.91–1.33)	0.303	1.2 (1.0–1.5)	0.065
Others	1.6 (1.42–1.88)	< 0.001	1.0 (0.8–1.2)	0.906
**Caste**				
Others caste	Ref		Ref	
Schedule Castes (SCs)	1.3 (1.05–1.54)	0.010	0.9 (0.7–1.1)	0.336
Schedule Tribes (STs)	1 .9 (1.57–2.23)	< 0.001	0.8 (0.6–1.0)	0.070
Other Backward Classes (OBCs)	0.9 (0.78–1.13)	0.517	0.6 (0.5–0.7)	< 0.001
**Wealth Index**				
Poorest	Ref		Ref	
Poorer	0.9 (0.74–1.19)	0.615	1.0 (0.8–1.3)	0.821
Middle	0.9 (0.73–1.13)	0.408	1.0 (0.8–1.3)	0.756
Richer	0.8 (0.66–1.02)	0.073	1.1 (0.8–1.4)	0.555
Richest	0.4 (0.36–0.56)	< 0.001	0.9 (0.6–1.2)	0.379
**Regions of India**				
North	Ref		Ref	
Central	4.0 (2.70–5.97)	< 0.001	3.6 (2.4–5.4)	< 0.001
East	10.0 (7.17–13.97)	< 0.001	9.3 (6.6–13.2)	< 0.001
Northeast	14.8 (10.70–20.48)	< 0.001	12.4 (8.7–17.6)	< 0.001
West	2.9 (1.88–4.45)	< 0.001	3.1 (2.0–4.8)	< 0.001
South	11.2 (8.05–15.77)	< 0.001	13.2 (9.3–18.7)	< 0.001

## Discussion

Our study presents the first national estimates of IKF in the paediatric population and includes several novel findings. First, there is a high prevalence of IKF among children and adolescents. This needs to be confirmed with repeated testing per KDIGO CKD criteria. Second, there were marked socio-demographic and demographic disparities in the prevalence of IKF. The estimated IKF prevalence of 4.9 (4.7–5.2)% in the CNNS sample amounts to around 49,000 (47,000–52000) cases per million population. Our estimates are similar to the prevalence observed in the Middle East and Southeast Asian countries [25]. Hospital-based epidemiological studies from Europe estimate the prevalence of pediatric CKD stages 2–5 between 30 and 100 per million age-related population. These data likely underestimate the true prevalence since only patients with overt CKD followed in a pediatric nephrology centre, are captured in the study and included in the numerator [18]. National-level estimation of CKD prevalence rates can be challenging in resource-constrained countries like India, as standard guidelines currently rely upon multiple eGFR values or sCr testing to confirm the diagnosis [26–30]. Still, many studies worldwide have used a single reading of eGFR to identify children with IKF rather than labelling them as living with CKD, which generates enough evidence to inform the policy [2, 31, 32].

We observed an age-wise non-linear increase in the prevalence of IKF, a trend similar to previous studies [33]. In part, the age-dependent decline may be an artefact of the Schwartz equation in children without CKD due to using a fixed *k* value of 0.413 for 2–18 years age groups. Females depicted a significantly higher mean eGFR, but there was no difference in the IKF prevalence between the two sexes, and unadjusted odds ratios were also non-significant. A multi-centric hospital-based registry of adult patients with CKD in India has shown a male preponderance; studies from other countries have documented a higher prevalence of CKD among females [5, 10]. One possible reason could be differential rates of blood testing during the survey to avoid the taboo associated with chronic diseases among females [9, 5, 34]. It is particularly important to identify CKD in females since it accentuates the risk of pregnancy-related complications like pre-eclampsia, premature birth, and small for gestational age or low birthweight babies, and consequently, fewer nephrons in their offspring who, in turn, will go on to develop CKD later in life [35].

Consistent with the published literature, obese participants depicted a higher prevalence of IKF [34, 38, 40] Wang et al. have shown that 24%–33% of all cases of kidney disease in the United States are associated with obesity across all age groups, including children and adolescents [35]. A prospective population-based study of 1.2 million adolescents depicted a 3.4 times higher risk of developing nondiabetic CKD in obese participants and a 19 times greater risk of developing diabetic ESRD, indicating a robust association between elevated BMI and CKD in adolescence [35]. We also observed a higher likelihood of having IKF in children and adolescents with stunting. The directionality of cause and effect cannot be established, as several factors could have influenced the development of both conditions, and CKD itself can contribute to or worsen stunting. Further, the proportion of obese children was relatively low and that of stunted high in this database, which reflects the overall high prevalence of undernutrition in India.

Our study documents several socioeconomic and regional disparities in the prevalence of IKF. Though IKF was non-significantly associated with IKF after adjusting for other variables, people from lower socioeconomic status depicted a higher prevalence of IKF, which is consistent with other studies [36, 37]. Available literature suggests that low socioeconomic status is linked with a higher likelihood of development of CKD due to its effect on health literacy, treatment-seeking behaviour and accessibility to services [38, 39]. This further highlights the need to study the role of socio-demographic disparities in differences in detail. The estimates for IKF depicted rural–urban differences, with higher prevalence and OR in rural areas. This is consistent with previous Indian studies [5, 40]. High prevalence in rural areas has been linked to poor environmental factors like poor water quality, worse nutrition and poorer access to healthcare [41]. Besides, there is a high use of pesticides and more secondhand smoke exposure among children in rural areas, which have been associated with CKD [42, 43]. The prevalence was high in the southern states and significantly lower in North India. This is consistent with previous general population studies showing a higher CKD prevalence in the southern states [44]. Geographic clusters with a high CKD burden have been reported, mostly from low- and lower-middle-income countries consisting of young males from agricultural communities who present with kidney failure without hypertension or proteinuria and are categorised as CKD of uncertain aetiology [45]. Such clusters have also been reported from India, most commonly from Andhra Pradesh, Tamil Nadu, Goa and Odisha. Environmental and genetic studies need to be done in those areas to gather more information for the predictability and progression of the condition. Previous studies attribute geographical disparities in the burden to poverty, poor sanitation, pollutants, water contamination, overcrowding, and known and unknown nephrotoxins (including heavy metals and plant toxins in indigenous remedies) that need further exploration through robust studies. Apart from the urban–rural divide, many castes and religious and cultural barriers exist. We observed that children from Islam and the “other” religions depicted a higher prevalence than Hindu children, but this association was non-significant on regression analysis. Such disparities, if any, should be further evaluated.

This study has a few policy implications. Despite the documented high and increasing prevalence, CKD has not received priority in public health programs globally, including in India. Further, it has primarily been considered a disease of the adult population, but the results emerging from the current study demand its prioritisation in the pediatric population as well. Given the emerging literature on the impact of low birth weight on the future development of CKD, maternal nutrition assumes importance in lowering the risk of CKD in the offspring. In light of the findings of this study the decision to include CKD in the National Programme for Non-Communicable Diseases (NP-NCD), formally known as the National Program for Prevention & Control of Cancer, Diabetes, Cardiovascular Diseases & Stroke (NPCDCS) program, is a welcome step. It is high time to raise awareness around pediatric kidney diseases through school health programs that will help early identification and management of affected children. Investment is also required in developing facilities to provide care to those with CKD, institute therapies to slow the progression of the disease and to ensure availability of kidney replacement therapies for those who may need them. Currently, there are few centres with experience in managing children with kidney diseases. More research is needed to develop a comprehensive understanding of the risk factors responsible for the development and progression of CKD. This includes the evaluation of socioeconomic determinants alongside the study of genetic, developmental, and environmental factors. This requires the setting up of cohorts, similar to the Indian Chronic Kidney Disease Cohort, which provides rich insights into CKD in the adult population in India [5]. Longitudinal studies will improve understanding of key risk factors for disease and inform policy on preventive strategies.

### Strengths and limitations

This study's strengths lie in the large number of participants enrolled nationwide using a robust methodology and the high response rate. This is the first analysis from India to depict eGFR estimates among the younger population using nationally representative data. However, this study is not free of limitations. The eGFR was determined only once, which could not allow us to make the diagnosis of CKD, which requires repeat testing after at least three months. Second, the study did not have data on albuminuria, another important marker of the presence of kidney disease. Also, the reliability of the Schwartz formula in detecting the early stages of CKD and predicting its course is generally poor. Its reliance on height leads to the generation of lower values in stunted children. Research towards finding better biomarkers of glomerular filtration rate, particularly those indicating early injury, is ongoing. Lastly, the accuracy of eGFR formulas has been disputed in many conditions like human immunodeficiency viral infection, chronic liver disease, cardiovascular disease, sarcopenia, hereditary disease (e.g., Fabry’s), and obesity [46]. The cross-sectional nature of the data limits us from making temporal causal associations. Also, we are limited in our ability to ascertain associations with the parameters collected in the survey. These limitations restrict out ability to fully explore the reasons behind some of the associations noted in this analysis. Finally, the findings of this study are only hypothesis-generating. For example, more work is required to understand better the geographic differences in kidney function and their drivers, explore the relationship between kidney function and stunting, and explore gender differences. Given that the kidney function is on a continuum, a strict eGFR cutoff (e.g. 60 ml/min/1.73m^2^)might rob the findings of nuances. It has been suggested that the eGFR threshold for making a diagnosis of CKD should be higher in the younger population. Certain variables, like the 15–19 year age group, depicted a significant change in OR on adjusted analysis, possibly due to the effect of confounders. However, we did not explore such confounding variables, which can be done in future studies. Despite these limitations, the information gathered during this study represents an advance in understanding the epidemiology of IKF and its determinants in India and a call to action for researchers and policy-making communities alike.

## Conclusions

For the first time, this study documents the prevalence of IKF among children and adolescents in India. These findings indicate the need for follow-up studies utilising internationally accepted methodologies and in accordance with the classification system to assess the prevalence of CKD accurately. CKD among adolescents is a dynamic and complex disease with unique factors that separate this population from adults. The impact of a CKD diagnosis in childhood and adolescence carries special significance. Data from this study provides new information to help develop the national strategy for CKD among children and adolescents in India and offer opportunities for international comparisons to further the goals of attaining optimal kidney health in the paediatric population.

### Supplementary Information

Supplementary Material 1.

## Data Availability

The ownership of the CNNS data lies with the Ministry of Health and Family Welfare, the Government of India, and the Population Council (India). Nevertheless, the author may be able to provide data upon a reasonable request, subject to the consent of the Population Council (India).
