# Supplementary material for: Prevalence and associated factors of impaired kidney functions among children and adolescents in India: insights from the Comprehensive National Nutrition Survey (CNNS) 2016-18
Source: BMC Pediatr. 2024 Jul 4;24:429. doi: 10.1186/s12887-024-04903-y (PMC11223285; doi:10.1186/s12887-024-04903-y)
Supplement: Supplementary file 1 — Supplementary Material 1. [file 12887_2024_4903_MOESM1_ESM.docx]

**Supplementary material**

**Supplementary material 1: Age-wise distribution of the participants included in the CNNS, 2016-18, India, based on their eGFR values.**

| **Age**  **(completed years)** |  | **EGFR values (***ml/min/1.73 m^2^***).** | | | | |
| --- | --- | --- | --- | --- | --- | --- |
|  | **<30** | **31-40** | **41-50** | **51-60** | **61-90** | **>91** |
| 5 | 0.72 | 1.68 | 3.19 | 3.39 | 20.39 | 71.58 |
| 6 | 1.13 | 0.85 | 2.63 | 3.38 | 20.8 | 72.84 |
| 7 | 0.84 | 1.05 | 1.92 | 3.41 | 18.16 | 75.19 |
| 8 | 1.09 | 0.9 | 1.52 | 2.86 | 20.88 | 73.85 |
| 9 | 0.68 | 0.51 | 1.43 | 2.2 | 19.28 | 76.93 |
| 10 | 0.53 | 0.34 | 2.68 | 2.15 | 15.37 | 79.78 |
| 11 | 0.84 | 0.64 | 2.11 | 1.82 | 17.23 | 77.99 |
| 12 | 0.48 | 0.45 | 1.4 | 2.49 | 17.56 | 78.37 |
| 13 | 0.93 | 0.58 | 0.65 | 2.55 | 19.9 | 75.87 |
| 14 | 0.51 | 0.24 | 2.12 | 2.5 | 25.47 | 71.06 |
| 15 | 0.43 | 0.5 | 1.46 | 1.89 | 31.41 | 66.11 |
| 16 | 0.77 | 0.28 | 1.52 | 3.94 | 32.4 | 63.22 |
| 17 | 1.14 | 0.3 | 2.31 | 3.29 | 36.5 | 57.69 |
| 18 | 0.73 | 0.75 | 2.58 | 3.47 | 43.04 | 51.4 |
| 19 | 0.98 | 0.6 | 2.15 | 2.64 | 40.11 | 55.85 |
| **Overall** | 0.81 | 0.73 | 2.0 | 2.85 | 23.41 | 71.4 |
